# Spontaneous Symptomatic Pseudoarthrosis at the L2-L3 Intervertebral Space with Diffuse Idiopathic Skeletal Hyperostosis: A Case Report

**DOI:** 10.1155/2013/497458

**Published:** 2013-11-05

**Authors:** Keiji Hasegawa, Hiroshi Takahashi, Yasuaki Iida, Yuichirou Yokoyama, Katsunori Fukutake, Ryo Takamatsu, Kazumasa Nakamura, Daisuke Suzuki, Wataru Shishikura, Shintaro Tsuge, Masayuki Sekiguchi, Akihito Wada

**Affiliations:** Department of Orthopaedic Surgery, Toho University School of Medicine, 6-11-1 Omori-Nishi Ota-ku, Tokyo 143-8541, Japan

## Abstract

Pseudoarthrosis at the intervertebral space in patients with ankylosing spondylitis has occasionally been reported, but symptomatic pseudoarthrosis at the intervertebral disc level is rare in patients with diffuse idiopathic skeletal hyperostosis (DISH). Here, we report a case of symptomatic pseudoarthrosis at the L2-L3 intervertebral space that was diagnosed based on clinical history. We first performed L1–L5 fixation, but back-out of the pedicle screw occurred in the early postoperative phase and may have been caused by a short fixation range and concomitant Parkinson's disease. However, the prognosis of the case was favorable after a second surgery. This case indicates that a fixation range of at least 3 above and 3 below is necessary for bone fracture of a thoracolumbar vertebra and pseudoarthrosis in patients with DISH.

## 1. Introduction

The ankylosed spine is prone to fracture after minor trauma due to its biomechanical properties. In patients with diffuse idiopathic skeletal hyperostosis (DISH), most fractures occur at the vertebral body, whereas in patients with ankylosing spondylitis (AS), fractures occur at similar rates at the vertebral body and intervertebral disc [1]. Pseudoarthrosis of the spine at the vertebral interspace in AS has been described [2–4], but this condition combined with painful motion at the site of pseudoarthrosis and an absence of a history of trauma is very rare in patients with DISH [5, 6]. Here, we report a case of DISH with spontaneous symptomatic pseudoarthrosis at the L2-L3 intervertebral space. The patient was treated successfully by posterior spinal instrumentation and bone grafting.

## 2. Case Report

A 76-year-old man presented with a 5-year history of low back pain. During this period, he had been repeatedly admitted to and discharged from a nearby hospital for lumbar pain. However, he had experienced particularly severe pain for 1 month without trauma and had difficulty walking and lying down due to pain in both lower extremities, and he was referred to our hospital. He had a history of Parkinson's disease and was receiving drug therapy in the Department of Neurology. He had a Hoehn-Yahr classification of III.

**Figure 1:**
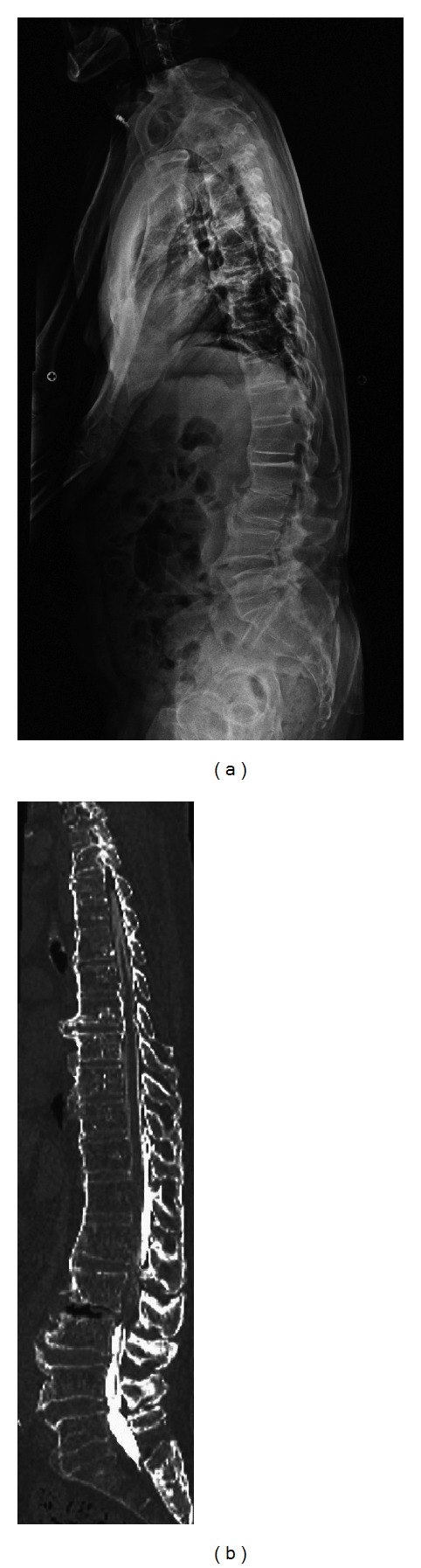
(a) Length in the standing position and lateral image in the standing position. (b) CT-MPR of the total length of the spine. The lateral X-ray image in the standing position showed no L2-L3 intervertebral space, but CT-multiplanar reconstruction images in the dorsal position showed a clearly opened L2-L3 intervertebral space.

**Figure 2:**
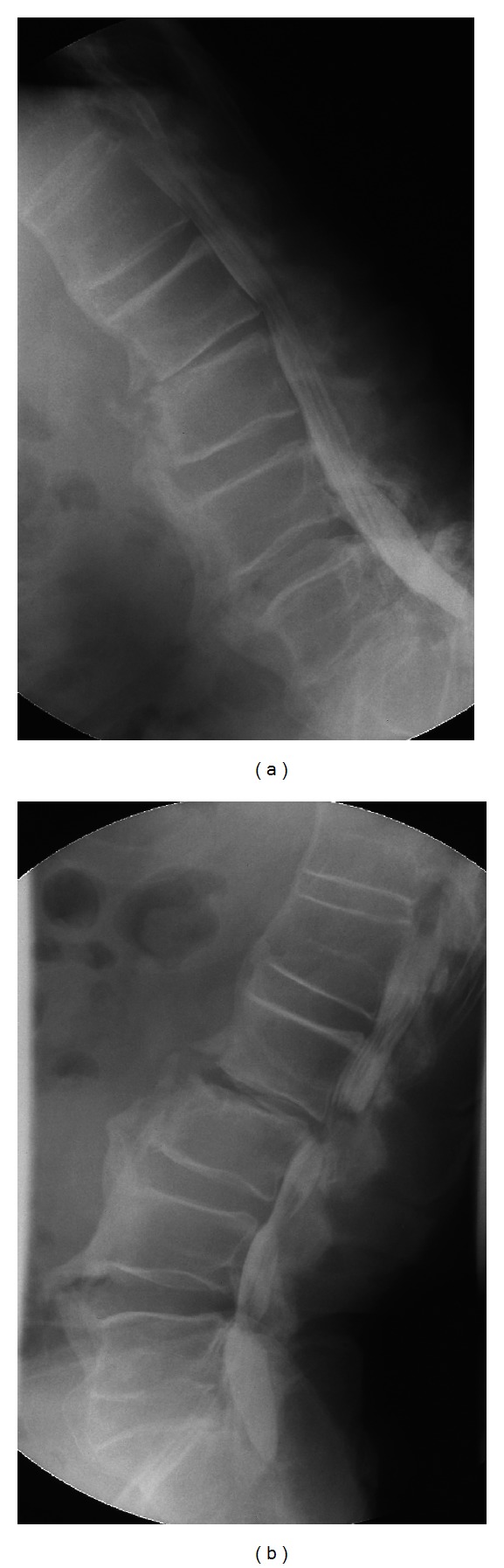
(a) Flexion and (b) extension images of the spine, showing instability at the L2-L3 intervertebral space and a stenosis of spinal canal is observed in the extension position.

At his first visit to our hospital, the range of motion of the trunk was severely restricted due to pain. A femoral nerve stretch test was positive and hypesthesia was noted in the bilateral L3 region. Laboratory studies showed that the white blood cell count, erythrocyte sedimentation rate, and C-reactive protein level were normal and that HLA-B27 was negative without findings of sacroiliac arthritis. Radiographs of the thoracic and lumbar spine showed flowing ossification along the anterior and lateral aspects of the Th2-L5 vertebral bodies, with disruption of ossific ridging and marked destructive changes at the L2-L3 intervertebral disc level (Figure 1). Myelography showed stenosis at the L2-L3 intervertebral space and flexion-extension radiograph indicated instability at this position (Figure 2).

**Figure 3:**
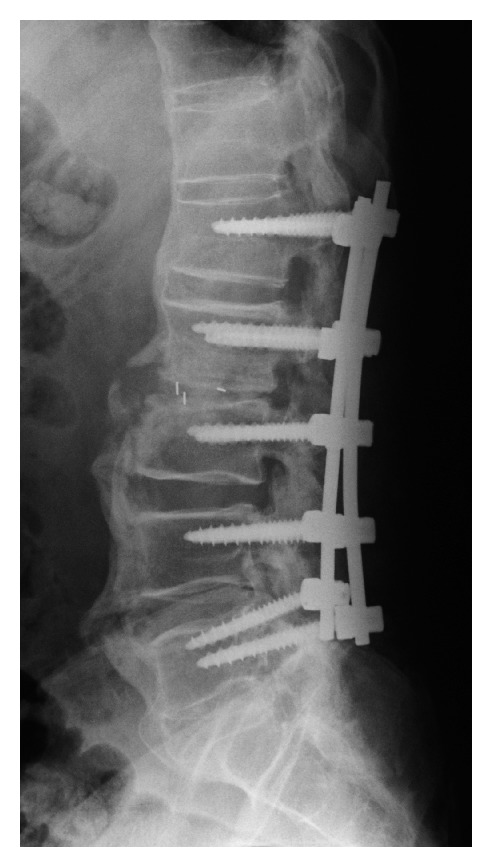
Lateral plain X-ray image obtained after the first surgery. In addition to posterior fusion at the L1–L5 intervertebral space, posterior lumbar interbody fusion at the L2-L3 intervertebral space was performed using a PEEK cage and local bone.

From these findings, we concluded that the unstable L2-L3 intervertebral space was spontaneous pseudoarthrosis occurring in the presence of DISH and that the motion at the site of pseudoarthrosis was the cause of the severe low back pain and thigh pain. We performed posterior fusion from the L1 to L5 levels with spinal instrumentation, in addition to posterior lumbar interbody fusion at the L2-L3 intervertebral space (Figure 3). The low back pain and thigh pain during motion disappeared immediately after surgery.

**Figure 4:**
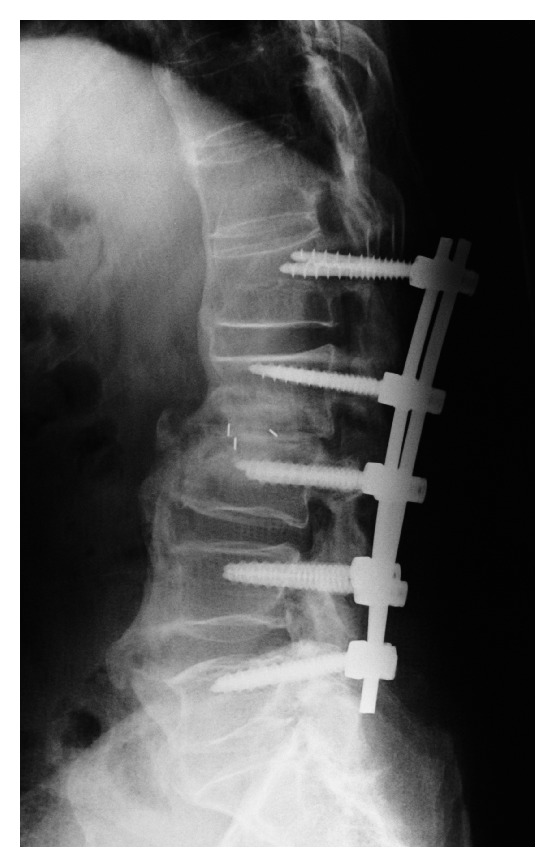
Lateral plain X-ray image obtained 10 weeks after the first surgery, showing loosening and back-out of the pedicle screw at L1-L2.

One week after surgery, the patient began walking exercises using a body trunk corset, but low back pain and thigh pain redeveloped in week 4. Radiograph findings suggested loosening and back-out of the pedicle screw inserted in the L1-L2 vertebral body. Fixation with a body trunk plaster cast was performed, but there was no improvement in symptoms and abnormal motion was apparent in the L2-L3 intervertebral space. Thus, a second surgery was performed 12 weeks after the initial surgery (Figure 4). The loosened pedicle screw at the L1-L2 vertebral body was removed and a new screw was inserted into the Th10, 11 and 12 vertebral body. Sublaminar taping was performed for each intervertebral space using polyethylene tape. Posterolateral fusion (PLF) was additionally performed for the L2-L3 intervertebral space using autologous iliac bone.

**Figure 5:**
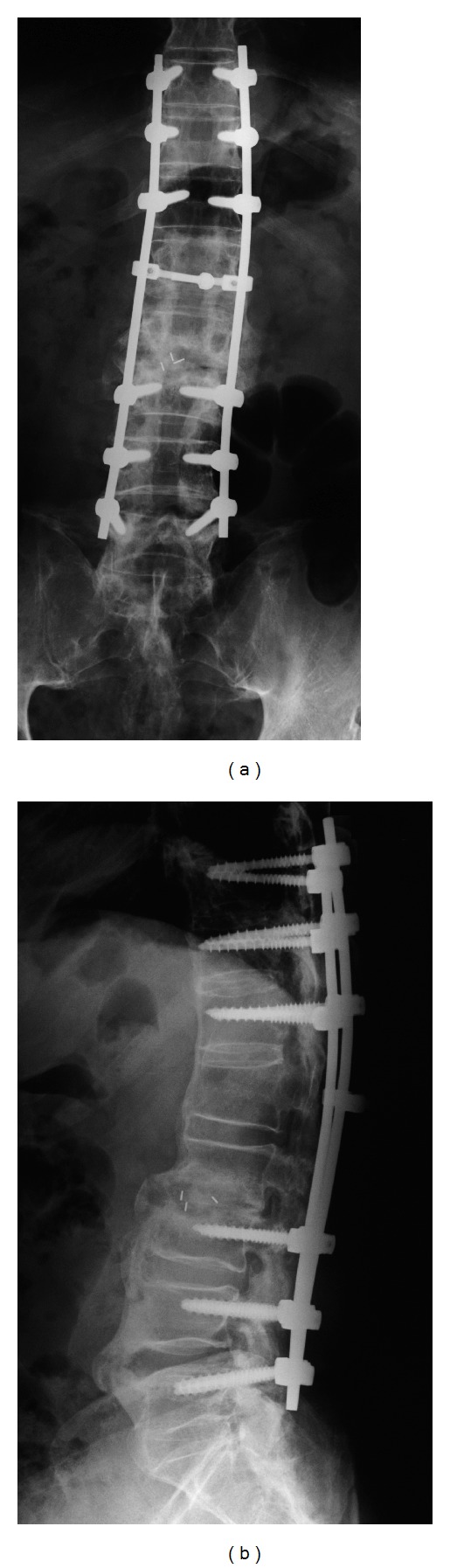
(a) Frontal and (b) lateral images obtained at 18 months after the second surgery, showing no loosening of pedicle screws and favorable bone adhesion in PLIF and PLF.

After the second surgery, low back pain and thigh pain were immediately improved. The second postoperative course was uneventful. The patient became able to walk and to lie down without pain. Plain radiographs taken 1.5 years after surgery showed rigid bony union at the L2-L3 intervertebral space (Figure 5).

## 3. Discussion

In 1971, Forestier and Lagier [7] coined the term ASH for the disease caused when an ankylosing spine develops due to hyperostosis-related changes mainly in the anterior longitudinal ligament. However, since ossification of ligaments of the extremities and their attachment sites occurs in many cases of ASH, Resnick and Niwayama [8] proposed the disease concept of diffuse idiopathic skeletal hyperostosis (DISH) to refer to the tendency of systemic bone proliferation and ossification of a longitudinal ligament. DISH is now used in more cases, compared to ASH, and has the following diagnostic criteria: (a) the presence of “flowing” calcification and ossification along the anterolateral aspects of at least 4 contiguous vertebral bodies with or without associated localized pointed excrescences at vertebral body-disc junctions; (b) relative preservation of disc height in the involved areas and the absence of extensive radiographic changes of “degenerative” disc disease, including vacuum phenomenon and vertebral body marginal sclerosis; and (c) the absence of apophyseal joint bony ankylosis and sacroiliac joint erosion, sclerosis, or bony fusion.

In DISH patients, unstable bone fracture of the spine may be caused by a slight external force. In such bone fracture, it is difficult to ensure bone union with conservative treatment, as seen in transverse fracture of long bones, and pseudoarthrosis or delayed dislocation is often observed. Furthermore, since such bone fracture occurs as low-energy trauma in many cases, the fracture may be overlooked at the first hospital visit and this may lead to an incorrect diagnosis and inappropriate treatment.

The fracture pattern is the striking difference between spinal fractures in AS and DISH. Whereas most fractures in AS traverse the disc space, the majority of fractures in DISH pass through the vertebral body [1]. This is because the depth and strength of ossification of the longitudinal ligament are high at an intervertebral disc level, while bone quality in the central vertebral body is low due to a stress shielding effect [9]. There have been several reports of pseudoarthrosis of the thoracolumbar spine at the vertebral interspace in AS [3, 4], including spontaneous pseudoarthrosis [2, 10]. However, pseudoarthrosis at a vertebral interspace is rare in patients with DISH. For bone fractures in ankylosing spine diseases such as AS and DISH, surgery is recommended instead of conservative treatment based on the incidence and prognosis of concomitant diseases [11, 12]. Solid fixation with posterior instrumentation is recommended as surgical treatment, with a suggested fixation range of at least 3 above and 3 below [13].

In our case, bone fracture might have occurred at the L2-L3 intervertebral space without trauma based on the disease history and then progressed to pseudoarthrosis. In the early postoperative phase after the first surgery, loosening and back-out of the pedicle screw occurred. This may have been due to the short fixation range on the head side and complications caused by Parkinson's disease. In patients with Parkinson's disease, the incidence of concomitant diseases and the rate of resurgery are high due to decreased bone quality and neuromuscular disorder caused by the disease [14, 15]. Thus, in our case, the fixation range should have been at least 3 above and 3 below in the first surgery, based on the history of Parkinson's disease.

## 4. Conclusion

We experienced a case of pseudoarthrosis at the L2-L3 intervertebral space that developed concomitantly with DISH in a patient with Parkinson's disease. Loosening and back-out of a pedicle screw occurred due to an insufficient fixation range in the first surgery, but a favorable prognosis was obtained after expansion of the fusion area and posterolateral fusion.
